# Iatrogenic Ureteral Obstruction During Transvaginal Oocyte Retrieval

**DOI:** 10.1590/S1677-5538.IBJU.2018.0692

**Published:** 2019-04-01

**Authors:** Ali Sami Gurbuz, Ali Cenker

**Affiliations:** 1 Deparment of Obstetrics and Gynaecology KTO Karatay University Medical Faculty Konya, Turkey;; 2 Novafertil IVF Center Konya, Turkey;; 3 Department of Urology, Akademi Meram Hospital Konya, Turkey

**Keywords:** Oocyte Retrieval, Ureter, Hematoma

## Abstract

Transvaginal oocyte retrieval is a crucial step in assisted reproductive technology. Various complications may arise during this procedure. Ureteral injury is a rare, but a serious complication in gynecological practice. During oocyte retrieval, ureteral injuries, detachment and obstruction can be seen, though rare. In this study, we will present ureteral obstruction that develops secondary to small hematoma, which mimics ovarian cyst torsion or ruptured ovarian cyst.

## INTRODUCTION

In vitro fertilization (IVF) and embryo transfer has been established as an integral part of assisted reproductive technology worldwide (1). IVF requires several steps in sequence, including stimulation of ovarian follicles, ultrasound-guided transvaginal oocyte retrieval, in vitro fertilization of the oocytes, and transfer of one or more embryos into the uterine cavity. Complications can occur during any of these steps and may rarely result in serious sequelae. The major complications of in vitro fertilization (IVF) include ovarian hyperstimulation syndrome (OHSS), multiple pregnancy, ectopic or heterotopic pregnancy (2). Transvaginal oocyte retrieval (TVOR) is the safest method for obtaining oocytes in in-vitro fertilization (3). It is a minimally invasive surgery; however, complications associated with surgical or clinical methods may be observed in this procedure. Vaginal bleeding, pelvic vascular injury, bowel injury, pelvic abscess, bladder and ureter injuries have been reported as complications. (4–6). In this study, we present a ureteric obstruction secondary to small hematoma forming around the right orifice on inferior surface of the bladder during transvaginal oocyte aspiration.

## CASE

The patient was a 35 years old woman with history of two early miscarriages and no long-term pregnancy. The physical examination revealed hirsutism and menstrual irregularity. In the anamnesis, an intramural myoma myomectomy operation was conducted through laparotomy; disc hernia and peptic ulcer were present. The patient was married for 7 years and underwent intrauterine insemination twice. Chromosome analysis performed on peripheral blood revealed a marker chromosome: 47XX+m. In ultrasonography examination, the patient was seen to have polycystic ovary appearance, but the uterus and endometrium were observed to be normal. Our case was coherent with polycystic ovarian syndrome (PCOS). On the second day of menstruation, a hormonal analysis was performed. The results were AMH- 6.4 ng / mL; Estradiol- 52.23 pg / mL; FSH- 6.16 mIU / mL; Lh- 21.22 mIU / mL; Prolactin- 10.35 ng / mL and TSH- 0.99 μIU / mL. Semen analysis was normal. Her body weight was 64 kg, and her BMI was 25 kg / m^2^. After a genetic consultation, polycystic ovary appearance, recurrent miscarriages and marker chromosome were taken into consideration, and IVF and preimplantation genetic diagnosis were planned.

## IVF ICSI procedure

An antagonist protocol with 187.5 IU rFSH (Gonal F, Merck, Turkey) was initiated. On the examination performed on day 6, 0.25 mg ganirelix per day (Orgalutran MSD Turkey) was initiated. She was stimulated for a total of 11 days. Due to OHSS risk, 0.2 mg triptorelin (Gonapeptyl Ferring Turkey) was used to trigger ovulation. Estradiol level on the day of triggering was 8999. The oocyte retrieval (OPU) was performed approximately 35 hours after the triggering.

**Figure 1:**
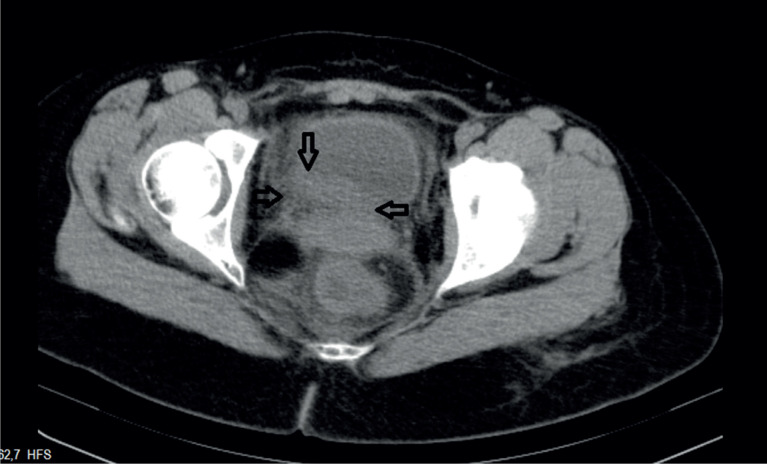
Hematoma on the inferior surface of the bladder.

**Figure 2:**
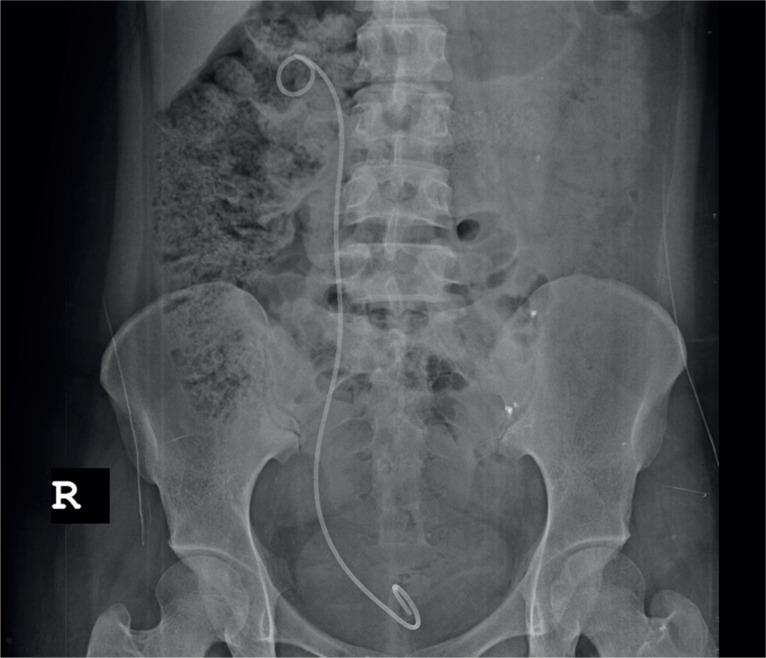
Double J stent, was inserted to right ureter to achieve ureteric passage.

Oocyte retrieval was performed by a subspecialist experienced in reproductive endocrinology and infertility using the standard transvaginal ultrasound-guided approach. The patient was prepped and draped in the dorsolithotomy position. The aspiration of ovarian follicles was carried out using a 17-gauge oocyte aspiration needle (Cook Medical Inc., Bloomington, IN) mounted on an ultrasonic transvaginal probe (Analogic Ultrasound Canada, Richmond, BC) with a needle guide and connected to a Cook Aspiration Unit (Cook Medical Inc., Bloomington, IN). 30 oocytes were collected, and IV fluid replacement and volume enhancers (Voluven Turkey) of approximately 2000 cc were given in the first 2 hours following the OPU operation. Meanwhile, severe right inguinal pain developed, and abdomen was tense. Transvaginal ultrasonography could not be performed due to pain. Abdominal ultrasonography was performed due to suspicion of ovarian torsion or ovarian cyst rupture. The right ovary measured 95 X 93 mm and the left ovary measured 104 X 96 mm. The vascularization was normal in both ovaries and no hematoma or mass was noted. The amount of free abdominal fluid was minimal. Painkillers (Diclofenac sodium and IV Paracetamol) were administered. Complete urinalysis was normal; no hematuria was detected. Pain persisted for 4 hours after the procedure, and therefore, the patient was referred to a tertiary referral hospital. According to the information received, a narcotic analgesic (Aldolan, Liba, Turkey) was administered and the patient was discharged 24 hours later since laboratory findings were the same, and pain was reduced. 40 hours after OPU operation, severe inguinal pain developed again, and the patient was hospitalized. The reason for pain could not be diagnosed. The pain intensified, and therefore, it was decided to perform exploratory laparotomy to exclude ovarian torsion or retroperitoneal hematoma. Pelvic tomography (CT) was performed on the third day prior to the laparotomy operation. Tomography revealed a hematoma, measuring 17 x 20 mm, and ureteral dilatation secondary to the hematoma on the inferior surface of the bladder, where the right ureter entered into bladder (Figure-1). When it was understood that this hematoma was the reason for ureteral obstruction, laparotomy operation was dropped. Cystoscopy was performed under general anesthesia and hematoma and edema were noted under the mucosa around right ureteral orifice. A 6F double J stent, measuring 26 cm, was inserted into the right ureter to achieve ureteric passage (Figure-2). The pain reduced dramatically after the procedure. Patient was discharged. The patient was examined again 21 days later, and it was noticed that hematoma had disappeared. A second cystoscopy was conducted, and catheter was removed.

Of the oocytes collected, 26 were mature. Fifteen oocytes were fertilized with intracytoplasmic sperm injection (ICSI). Four embryos reached the blastocyst stage and trophectoderm biopsy was performed. Each embryo was separately cryopreserved. Preimplantation genetic screening was performed by the Next Generation Sequencing method. Two embryos were normal; one of the four had multiple chromosomal anomalies, and the other had mosaic trisomy 15. Two normal embryos were transferred after preparing the endometrium, and two healthy babies were delivered by cesarean section after a healthy pregnancy period.

## DISCUSSION

TVOR is a safe, simple and effective method. Vaginal bleeding and pelvic infection are the most common complications that occur after oocyte retrieval by ultrasound (7). Ureteral injuries are considered as serious complications of all gynecological cases; a literature review shows that ureteric complications are rare after TVOR (8). Ureteral injuries are quite rare due to its anatomical position (9). Ureteral injury risk can occur in conditions leading to anatomic irregularities, such as endometriosis, previous operations, and pelvic inflammatory diseases and due to distortion that occurs when pressure is applied by the transvaginal ultrasound probe (10).

Nausea, vomiting and pelvic pain are observed in ureteral complications that developed after oocyte retrieval, and also hematuria, abdominal distension, and vaginal discharge are detected. Transvaginal ultrasound, intravenous pyelography (IVP), or CT are recommended for diagnosis (11, 12).

The main problem in our case was the presence of polycystic ovary syndrome and hyperstimulation due to this syndrome. During TVOR, inserting a needle through the side wall of the vagina and pulling it out of the ovary were performed several times with aspiration needles in order to aspirate all follicles in the hyper-stimulated ovary. Meanwhile, hematoma developed over time on the bladder wall and around the ureteral orifice with the injury of small vessels under the bladder. We think that pelvic distortion having developed secondary to myomectomy may also have played a role in hematoma. In our case, no symptom was observed except severe right inguinal pain. Also, we focused on ovarian torsion in the acute phase, since hematuria was not present in our case, and the hematoma was not observed on the backside of the bladder, because only abdominal ultrasonography was performed. If TVUSG, CT, or IVP had been performed in the first few hours, hematoma or distal obstruction in the ureter could have been detected.

In a similar case, a patient from India had hematoma on the uterus adjacent to right ureter. However, hematoma was evident, and the clinical picture was improved after placement of ureteral stent (6).

When a literature review is performed, it may be observed that 4 cases out of 14 had ureteral obstruction. Of two cases with ureteral stent placement, one required ureteral re-implantation, and the other required nephrectomy secondary to renal failure (11, 13–15). Percutaneous nephrostomy has also been performed in the ureteral injury for treatment (16). In our case, ureteral stent placement was performed, and the clinical picture improved over time once hematoma was reabsorbed.

## CONCLUSIONS

Ureteral injury is a very rare complication. If there are no findings such as nausea, vomiting, abdominal distension, and hematuria, it is difficult to diagnose. For early diagnosis, a CT or cystoscopy should be performed on patients suffering from severe pelvic pain to exclude hematoma or ureteral obstruction. During treatment, placing a temporary stent will dramatically reduce symptoms if the ureter is intact.
